# Takotsubo Syndrome and Coronary Artery Disease: Which Came First—The Chicken or the Egg?

**DOI:** 10.3390/jcdd11020039

**Published:** 2024-01-26

**Authors:** Mihail Celeski, Annunziata Nusca, Valeria Maria De Luca, Giorgio Antonelli, Valeria Cammalleri, Rosetta Melfi, Fabio Mangiacapra, Elisabetta Ricottini, Paolo Gallo, Nino Cocco, Raffaele Rinaldi, Francesco Grigioni, Gian Paolo Ussia

**Affiliations:** 1 Fondazione Policlinico Universitario Campus Bio-Medico, Via Alvaro del Portillo, 200, 00128 Roma, Italy; mihail.celeski@unicampus.it (M.C.); valeriamaria.deluca@unicampus.it (V.M.D.L.); giorgio.antonelli@unicampus.it (G.A.); v.cammalleri@policlinicocampus.it (V.C.); r.melfi@policlinicocampus.it (R.M.); f.mangiacapra@policlinicocampus.it (F.M.); e.ricottini@policlinicocampus.it (E.R.); p.gallo@policlinicocampus.it (P.G.); n.cocco@policlinicocampus.it (N.C.); r.rinaldi@policlinicocampus.it (R.R.); f.grigioni@policlinicocampus.it (F.G.); g.ussia@policlinicocampus.it (G.P.U.); 2 Research Unit of Cardiovascular Sciences, Department of Medicine and Surgery, Università Campus Bio-Medico di Roma, Via Alvaro del Portillo, 21, 00128 Roma, Italy

**Keywords:** takotsubo syndrome, coronary artery disease, stress-induced cardiomyopathy, acute coronary syndrome, myocardial stunning, microvascular dysfunction

## Abstract

Takotsubo syndrome (TTS) is a clinical condition characterized by temporary regional wall motion anomalies and dysfunction that extend beyond a single epicardial vascular distribution. Various pathophysiological mechanisms, including inflammation, microvascular dysfunction, direct catecholamine toxicity, metabolic changes, sympathetic overdrive-mediated multi-vessel epicardial spasms, and transitory ischemia may cause the observed reversible myocardial stunning. Despite the fact that TTS usually has an acute coronary syndrome-like pattern of presentation, the absence of culprit atherosclerotic coronary artery disease is often reported at coronary angiography. However, the idea that coronary artery disease (CAD) and TTS conditions are mutually exclusive has been cast into doubt by numerous recent studies suggesting that CAD may coexist in many TTS patients, with significant clinical and prognostic repercussions. Whether the relationship between CAD and TTS is a mere coincidence or a bidirectional cause-and-effect is still up for debate, and misdiagnosis of the two disorders could lead to improper patient treatment with unfavourable outcomes. Therefore, this review seeks to provide a profound understanding of the relationship between CAD and TTS by analyzing potential common underlying pathways, addressing challenges in differential diagnosis, and discussing medical and procedural techniques to treat these conditions appropriately.

## 1. Introduction

Takotsubo Syndrome (TTS) is a reversible cardiomyopathy defined by an abrupt transient left ventricular (LV) dysfunction that can be linked to potentially fatal conditions such as arrhythmias and cardiogenic shock (CS) [1].

Since its first description in 1990 in Japan by Sato et al., TTS has become a topic of great interest to scientists and physicians. It has also been referred to by various names, including apical ballooning syndrome, broken heart syndrome, and stress cardiomyopathy [1]. However, as some investigations have shown, stress-induced heart injury (SIIH) was documented as early as the 1970s, when there were no techniques available for the in vivo identification of stress cardiomyopathy in human beings [2,3]. Nevertheless, the high frequency of TTS, which is still misdiagnosed, makes it necessary to standardize knowledge and apply definite diagnostic criteria. In this regard, the first diagnostic criteria were introduced by Abe et al. in 2003 [4]. Subsequently, several diagnostic criteria, including the Mayo Clinic criteria, and the most recent International Takotsubo Diagnostic Criteria (InterTAK Diagnostic Criteria) were proposed to facilitate TTS diagnosis [1,5].

Nevertheless, recent studies have suggested that TTS and coronary artery disease (CAD) may coexist, challenging the previous criteria and the concept that the presence of CAD inevitably excludes TTS diagnosis. The coexistence of both conditions may have important clinical and prognostic repercussions and should be considered during the process of diagnostic differentiation and therapeutic planning. Indeed, clinical manifestations of TTS include signs and symptoms indistinguishable from acute coronary syndromes (ACS), such as electrocardiographic abnormalities, increased biomarkers, clinical presentation, and temporary changes in the echocardiography-detected LV myocardial kinetics [1]. Additionally, TTS affects 1–3% of all patients admitted to the emergency departments with suspected ST-segment elevation myocardial infarction (STEMI), and the incidence rises to 5–6% in women with probable ACS [6,7]. Despite the early belief that TTS was a benign disease, earlier research already demonstrated that the disease’s mortality and morbidity rates were similar to those of ACS [8]. On the other hand, patients who present contemporary CAD and TTS may have even worse prognoses and clinical outcomes [9]. It is currently unclear if a causal association exists between CAD and TTS, but incorrect diagnosis could bring detrimental consequences. As a result, the heart tissue and microcirculation have become the primary focus of recent investigations that aimed to explore the underlying mechanisms of TTS [10]. Although sympathetic stimulation, microvascular dysfunction, heart cell metabolism change, and inflammation have been widely investigated, other mechanisms such as coronary thrombus’s spontaneous thrombolysis, coronary vasospasm, and transient ischemia might play a role [11]. However, whether coronary lesions are a cause–effect or bystander in the context of TTS, and which are the main mechanisms behind this association, is still unclear. 

Therefore, examining probable shared driving mechanisms, discussing difficulties in differential diagnosis, and reviewing medical and procedural strategies to treat both conditions effectively, this review aims to provide a thorough comprehension of the association between TTS and CAD.

## 2. Methods

We conducted a comprehensive review, without regard to language restrictions, of all manuscripts published between 1974 and 2023 that were identified by search in Pubmed, PMC, and Cochrane. TakoTsubo Syndrome, Tako-Tsubo Cardiomyopathy, Apical Ballooning Syndrome, Stress induced heart injury, Left Ventricular Apical Ballooning Syndrome, Stress Cardiomyopathy, Transient Apical Ballooning Syndrome, Coronary artery disease and Takotsubo Syndrome, microvascular dysfunction, and pathophysiology of Takotsubo Syndrome, were the main Mesh terms and keywords. In order to perform additional investigation, we also browsed through the references of the publications that were obtained. The majority of the articles that were taken into consideration were expert consensus, meta-analyses, clinical investigations, case reports, and narrative and systematic review articles. It was determined to omit any manuscript that failed to respond to our questioning. The following are the main sections in which we arranged the search and result description: emerging concepts of Takotsubo syndrome pathophysiology, coexistence of Takotsubo and coronary artery disease—coincidence or cause and effect, Takotsubo triggered by ischemia—the chicken–egg paradox, Takotsubo and coronary syndromes—to differentiate or co-treat, coronary artery disease and Takotsubo synergism—possibility of cotreatment, conclusions and future directions.

## 3. Emerging Concepts of Takotsubo Syndrome Pathophysiology

The exact mechanisms underlying TTS are still unknown. Despite extensive research, a comprehensive knowledge of this condition, its underlying pathogenesis and pathophysiology, is still lacking. 

Nearly 50 years ago, Johansson et al. proposed the expression “stress-induced cardiopathy” to characterize the SIIH with particular myocardial morphologic and electrocardiographic alterations, including necrosis, that resulted from immobilization (emotional stress) on pigs [2]. This led to further research into the pathophysiology of this condition. Up until the term “stress cardiomyopathy” was coined in the 1990s, numerous research had shown that SIIH could occur in both humans and animals [3].

Most research efforts have been directed toward investigating the potential role of adrenergic hyperactivity and catecholamine increases in the onset of this syndrome. Indeed, TTS can be brought on by both positive and negative emotions causing a strong release of catecholamines [12]. The brain’s cognitive regions, particularly locus coeruleus, trigger the hypothalamic–pituitary–adrenal axis in reaction to stress, increasing the release of epinephrine and norepinephrine [13]. Catecholamines and stress-related neuropeptides are generally released systemically but may also be delivered locally. In fact, it was demonstrated that patients with TTS have higher concentrations of norepinephrine in their coronary sinuses, which suggests a greater release of catecholamines from the cardiac cells [14]. Accordingly, cardiomyocytes’ internal signal transmission pathway may change as a result of elevated catecholamine levels. The G(s) protein signalling is replaced by G(i) protein signalling, which prevents excessive activation of myocytes and reduces contractility via the beta2-adrenoceptor (beta2-AR). This appears to be particularly noticeable in the apical myocardium, which has the highest density of beta-adrenoceptors [15]. Many experimental studies on adrenergic receptors showed interesting results. It has been shown that the negative inotropic effect caused by beta2-AR activation in conjunction with Gi-proteins is eliminated by beta2-AR blockage and the application of a pertussis toxin-based Gi-protein inhibitor; however, the effect remains after the administration of a specific beta1-AR antagonist. Further experimental research demonstrated that cardiac contractility alterations and irregularities in wall motion can be caused by large concentrations of epinephrin administration or xylazine, an alfa2-AR agonist, which eliminates the pertussis toxin [3]. Nevertheless, a recent study conducted by Kurbatov et al. that studied the role of adrenergic receptors, muscarinic receptors, and hormones in SIIH provided new findings. Through immobilization stress on male and female Wistar rats, they showed that emotional stress promotes the release of catecholamines, corticosterone, and aldosterone, and induces myocardial injury and contractility decrease, mimicking the human TTS. They also showed that activation of beta1-AR plays a key role in SIIH, while beta2-AR stimulation has a cardioprotective effect, preventing SIIH. They also suggest that more receptors may be involved in the SIIH, and others, such as muscarinic receptors, in the regulation of cardiac stress tolerance [16]. On the other hand, catecholamines that approach the heart through sympathetic nerves and are released directly into the myocardium may have greater cardiotoxic effects compared to those delivered by circulation [17]. Consequently, norepinephrine spillover can reduce myocyte viability, leading to contraction band necrosis—a histological characteristic associated with TTS [18].

Moreover, patients with neurological and psychiatric disorders have a higher prevalence of TTS. The autopsy reports of individuals who passed away unexpectedly from physical violence were published in the study by Cebeli et al., demonstrating that all victims of violence had heart myofibrillar degeneration, which is only possible as the consequence of psychological trauma [19]. The core autonomic network of the brain has been shown to be altered in more recently functional magnetic resonance imaging investigations in patients suffering from this syndrome, underscoring the significance of the brain–heart axis in the development of TTS [20]. Recent research has also revealed anomalies in the sympathetic nervous system and emotion-related brain regions’ activity and functional organization. The brain stem, hippocampus, and basal ganglia exhibit notable alterations with increased flow both during the acute phase and at follow-up following TTS [21]. Nevertheless, the brain–heart axis is still an intriguing subject that must be investigated extensively.

However, in a notable percentage of patients, identifying a plausible stressor remains unattainable, and other pathophysiological mechanisms have been proposed as potential contributors to the onset of TTS.

Firstly, the high prevalence of TTS in postmenopausal women suggests a hormonal influence, represented by the estrogen level decline after menopause. Estrogens can affect vasomotor tone by upregulating nitric oxide synthase in the endothelium. Indeed, in ovariectomized female rats subjected to immobilization stress, a study revealed that estrogen supplementation can considerably raise blood pressure and ameliorate stress-induced heart dysfunction [22]. Additionally, there is proof that in perimenopausal women, estrogens can lessen the sympathetic response to mental stress and mitigate catecholamine-mediated vasoconstriction [1]. 

Furthermore, although Mendelian inheritance has not been demonstrated, familial cases of TTS suggest a genetic predisposition. Considering the role of catecholamines in triggering this syndrome, polymorphisms of adrenergic receptors have been extensively studied. While beta2-receptor polymorphisms Gln27Glu [homozygous glutamine (Gln)/Gln] are more commonly discovered in healthy controls, beta1-receptor polymorphisms Arg389Gly [homozygous arginine (Arg)/Arg] are more typically detected in TTS patients [1]. In addition, an L41Q polymorphism of G protein-coupled receptor kinase 5 (GRK5), one of the more frequently occurring isoforms in the heart, has been found to be a susceptibility factor in individuals with TTS, emphasizing the role of G protein-coupled receptor signaling desensitization and downregulation in TTS [23]. 

However, increasing evidence exists claiming that the acute phase of TTS is characterized by cardiac inflammation and metabolic and energetic impairment. 

Higher systemic proinflammatory cytokine levels, alterations in the distribution of monocyte subsets, and macrophage myocardial infiltration all contribute to increased inflammation in the myocardium [24]. Since these alterations were seen to last for at least five months, a low-grade chronic inflammatory disease may be present in TTS patients [24]. It may also have prognostic implications, as higher levels of pro-inflammatory (IL-2, IL-6) and anti-inflammatory (IL-10) interleukins at admission are linked to a higher likelihood of adverse events during follow-up in TTS patients [25]. Furthermore, an elevation in tumor necrosis factor alfa (TNF-α), IL-6, C-reactive protein, and chemokine (C-X-C motif) ligand 1 (CXCL1), which are components of the systemic inflammatory response in TTS, implies that TTS induces myocardial inflammation and edema [3]. Post-mortem examination of hearts from patients who died during the acute phase of TTS shows that rather than reparative type (M2), the majority of the macrophages are proinflammatory (M1) type [26]. Compared to the similar stages observed in patients with acute myocardial infarction (AMI), the presence of M1 macrophages and the persistence of the intermediate (CD14++CD16+) monocyte subpopulation over the course of a 5-month observation period after acute TTS strongly suggest a more proinflammatory and less regenerative state in TTS [26]. However, more research is required to clarify the involvement of inflammation in the pathophysiology of TTS and the determination of clinical manifestation and outcome.

Several data point to the possibility that oxidative stress brought on by exogenous or endogenous sources may be a prevalent characteristic in the pre-acute stage of TTS. However, the acute phase of TTS is characterized by a high oxidative status, which may lead to further myocardial cell damage and endothelial dysfunction. These effects can influence the synthesis of vasoactive molecules and limit tissue perfusion at the microcirculatory level [27]. 

Moreover, according to recent research, metabolic and energetic impairment is also present throughout the acute phase of TTS and is followed by partial recovery [24]. Reduced synthesis of Kreb’s cycle intermediates and adenosine triphosphate results from an increase in myocardial glucose intake combined with a decrease in the available glycolysis metabolites, giving rise to a different metabolic profile in TTS than patients with AMI [28]. In a recent study, serum metabolic profile analysis showed that TTS is associated with a substantial rise in the plasmatic concentrations of ketone bodies, 2-hydroxybutyrate, and markers associated with fatty acid metabolism, which leads to endothelial dysfunction through the production of oxygen radicals and vascular inflammation [29,30,31]. Additionally, TTS patients have decreased concentrations of several amino acids, which are correlated in a proportionate way with LV systolic function [29,32].

**Figure 1:**
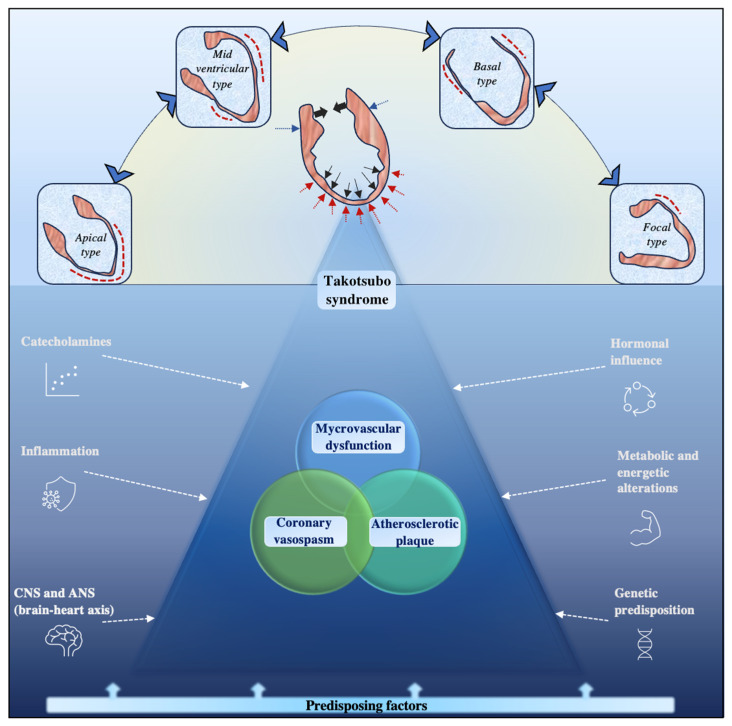
Main pathophysiological mechanisms in Takotsubo Syndrome. CNS = central nervous system; ANS = autonomic nervous system.

Despite the different points of view and proposed mechanisms, all the above-mentioned pathways generally lead to myocardial, endothelial, and microvascular alterations. Pathophysiological mechanisms in TTS are schematically represented in Figure 1. It is not by chance that a high percentage of patients with TTS also have obstructive and non-obstructive CAD [9]. Indeed, several pathogenetic mechanisms of TTS have been proposed in this setting. Firstly, it has been hypothesized that coronary artery multivessel spasm, which is commonly linked to an adrenergic surge, may be a pathogenic factor in TTS. Provocative testing with acetylcholine early after a TTS episode can replicate a diffuse, transitory spastic sub-occlusion/occlusion of epicardial arteries and critical ischemia in about 21% of patients, according to the theories about aberrant coronary vasomotion [33]. Indeed, stress, a trigger for TTS in most cases, can induce endothelial dysfunction through endothelin-A receptors [34]. Since none of the previously mentioned evidence can provide an explanation for the non-apical types of TTS, some authors have hypothesized that vasospasm and preexisting endothelial dysfunction are likely to be the driving forces behind TTS development [35]. Moreover, it has also been proposed that myocardial bridging may play a role in the development of TTS [36].

Among the pathogenetic hypotheses, the rupture of an atherosclerotic plaque as a cause of TTS has also been proposed [37]. An aborted myocardial infarction seems to be reasonable in such cases. Studies using intravascular imaging showed that in the great majority of these individuals, there is no evidence of ruptured plaques, active erosions, or intracoronary thrombi, suggesting no causative relationship between plaque complication and TTS [38,39]. In any case, these studies are small, and not all the patients included in them had routine use of intravascular imaging techniques. Therefore, this hypothesis cannot be easily excluded. The underlying factors of CAD and TTS association are described in more detail later. 

On the other hand, according to recent findings, microvascular dysfunction is given special consideration while discussing TTS physiopathology since microcirculation regulates coronary blood flow in response to metabolic, mechanical, and neural influences. In addition, catecholamines also exert vasoconstrictive effects on microvessels where alpha1-receptors predominate. This is the basis for the hypothesis of coronary microvascular dysfunction (CMD) as the main cause of TTS [40,41]. The increase in microvascular reactivity in TTS patients is mediated by the activation of the sympathetic nervous system and translates into myocardial stunning. Even in this case, women are more likely to have CMD than males, especially when they are postmenopausal, as a significant predisposing factor increasing the coronary microcirculation’s sensitivity to adrenergic stimulation is ovarian hormone deficiency [42]. It appears likely that blood viscosity, which catecholamines may also raise, is a major factor in the etiology of microcirculatory dysfunction [3]. Moreover, in the early phase of TTS, clinical investigations employing serial Doppler transthoracic echocardiography or positron emission tomography (PET) scans showed decreased microvascular blood flow and coronary flow reserve, indicating myocardial dysfunction as a potential mechanism [43]. The microvascular involvement has also been confirmed by evaluating the vascular response to intravenous adenosine injections. This test temporarily enhances myocardial perfusion, LV ejection fraction (LVEF), and wall motion score index (WMSI) during the acute phase of TTS [44]. On the other hand, in a study conducted by Redfors et al., a myocardial contrast echocardiography was used to measure microcirculation in rats that had SIIH brought on by beta-AR agonist isoproterenol injection. In the early stages of stress-induced damage in this rat model, apical perfusion was not compromised [45]. However, a very recent experimental finding provides new notions. The CMD phenotype is imitated by the loss of vascular potassium Kv1.5 channels, which link myocardial metabolism to coronary blood flow. Using transaortic constriction as a stress trigger, Dong et al. investigated wild-type Kv1.5−/− and TgKv1.5−/− (Kv1.5−/− with inducible smooth muscle-specific expression Kv1.5 channels) mice. In comparison to control groups, they found that in Kv1.5−/− animals, transaortic constriction resulted in systolic apical ballooning and decreased LV apex myocardial blood flow. The apex and base of the LV were restored to normal function and perfusion by using a vasodilator agent (chromonar) to increase myocardial blood flow, or by inducing smooth muscle expression of Kv1.5 channels in TgKv1.5−/− mice [46]. Accordingly, it has recently been suggested that TTS may represent the tip of the iceberg in the clinical spectrum of coronary microvascular dysfunction [10]. Indeed, only a moderate stimulus may be enough in high-risk people with greater sympathetic tone and vasomotor dysfunction (postmenopausal status, depression, etc.) to cause microvascular ischemia and subsequent myocardial stunning [47].

However, it is imperative to accept that the underlying pathophysiology of TTS is still poorly understood and is a topic of continuous scientific debate. 

## 4. Coexistence of Takotsubo and Coronary Artery Disease—Coincidence or Cause and Effect

Although the lack of significant obstructive CAD or angiographic evidence of plaque rupture represented a key component of the Mayo criteria for diagnosing TTS [48], several recently published case series have cast doubt on the idea that the presence of CAD automatically rules out the diagnosis of TTS [49,50]. Moreover, many early studies on TTS excluded individuals with coexisting CAD; therefore, its prevalence in those patients was underestimated. Nonetheless, recent TTS research reported various association rates between CAD and TTS, with a percentage ranging widely from 10% to 60% [51,52,53,54,55,56]. While Takotsubo Italian Network (TIN) found that about 10% of the enrolled individuals with TTS were also affected by CAD [57], the database of the Nationwide Inpatient Sample (NIS–USA), which analyzed nearly 25,000 patients, revealed that almost 45% of TTS patients had concomitant CAD [58]. This wide percentage range and significant variation can be explained by the fact that most of the studies either included small numbers of participants or employed different TTS diagnostic criteria. Patients with TTS may not only show modest coronary lesions, but they may also have coexisting severe coronary stenosis that may occasionally act as a trigger of the TTS [55,59]. Even though the left anterior descending (LAD) artery is most affected, there may also be the presence of other diseased coronary vessels or even a multi-vessel involvement [60]. Indeed, in the most recent study conducted by Napp et al., among 1016 patients that met the most recent InterTak Takotsubo diagnostic criteria (female sex, emotional stress, physical stress, non ST-depression, acute, former, or chronic neurological or psychiatric disorder, prolonged QTc time), there was an unexpectedly high rate of coexisting CAD (64.2%) [9]. One-third of the patients had coronary stenoses more than 50%; notably, some of them experienced acute coronary occlusion [9]. This is crucial, because the underdiagnosis of TTS may be significantly exacerbated by the incomplete understanding of the coexistence of obstructive and non-obstructive CAD and TTS. 

Although TTS was previously considered to be a benign condition, cardiogenic shock, malignant arrhythmias, or cardiac arrest might aggravate the clinical course of TTS [61,62,63]. The incidence of major adverse cardiac and cerebrovascular events is reported to be 10% per patient-year, whereas the death rate is 6% [51]. Notably, in TTS patients, the existence of CAD is significantly associated with a further worsening of prognosis. Indeed, the coexistence of CAD was shown to be associated with higher rates of shock, ventilation, and overall death [9]. Moreover, according to Bill et al., individuals with TTS and concomitant CAD had a greater 2-year all-cause mortality rate than those with isolated TTS [54]. 

All this information is fundamental, as misdiagnosis may lead to taking the wrong therapeutic strategy, which could become detrimental for the patient. 

CAD and TTS coexistence can be partially explained by the commonly shared risk factors. Studies revealed that patients with TTS are mostly elderly and women, and they are also affected by hypertension (in near half of the patients), dyslipidemia, diabetes mellitus (DM), and near one-quarter have smoking habits [64,65]. Indeed, atherosclerosis lesions have been reported in many TTS patients, raising important prognostic implications [66,67]. Moreover, this encourages a more patient-centered approach to therapy, emphasizing the value of tailored risk assessment and management plans that take into consideration the comorbidities and risk profiles of TTS patients. Nonetheless, it is noteworthy that compared to individuals with DM without TTS, patients with ACS, and the overall population, TTS patients have a lower prevalence of DM [68,69]. The lower than expected prevalence of DM in individuals with TTS suggests that DM prevents the onset of TTS, while it has a clinically improving effect after the TTS development (a phenomenon known as the “diabetes paradox”) [70]. In fact, Ahuja et al. discovered that the complicated DM may be protective in the near term for TTS patients’ outcomes, presumably as a result of a slowed catecholamine-mediated reaction [69]. But further research is needed on this topic. Furthermore, alterations in metabolism and inflammation might be additional common underlying processes in both conditions. Through oxidative damage in the atherosclerotic process and inflammation activation, pro-inflammatory macrophages and monocyte proliferation promote inflammatory responses [71]. On the other hand, these protagonists are the main players in the development of TTS, as was already mentioned. Destabilization and progression of atherosclerotic plaque are critical factors in the beginning and progression of coronary artery disease, resulting from persistent inflammation [71]. The TTS-causing inflammatory process may trigger plaque instability, leading to ischemic episodes. Moreover, according to recent studies, the atherosclerotic process is influenced by metabolic reprogramming that involves important metabolic pathways like amino acid metabolism, fatty acid oxidation, and glycolysis [72]. These pathways have also been shown to play a role in the development of TTS, as described previously. Finally, the development and evolution of both CAD and TTS can be influenced by endothelial dysfunction and microvascular involvement [43,73].

Once coexistence between TTS and CAD has been established, multiple possible scenarios are possible. The first situation is that coronary artery atherosclerosis could be a concomitant incidental finding, a ‘’bystander’’ that existed before the development of the TTS. As mentioned before, the particular epidemiology of TTS, which commonly affects older postmenopausal women, and the high incidence of common cardiovascular (CV) risk factors and shared novel underlying mechanisms such as inflammation and metabolic changes, may explain this connection [74]. In the study conducted by Either et al., nearly 15% of the TTS patients had important atherosclerotic plaques, mostly seen in either the LAD artery or both the LAD and the left main coronary artery, with one-third having several types of plaque [38]. They did not find any plaque rupture, thrombosis, or coronary dissection using optical coherence tomography (OCT), sustaining the hypothesis that CAD and TTS may coexist without sharing a direct causal connection [38]. Furthermore, some other studies showed no evidence of plaque thrombosis or ulceration, nor intravascular ultrasound (IVUS) evidence of coronary spasm or distal embolization to explain the myocardial stunning and apical ballooning, suggesting that CAD is usually a bystander in these individuals [75,76]. Nevertheless, in these studies, not all patients had IVUS examinations. Moreover, an ischemia-triggered TTS cannot be easily excluded only by using intra-coronary imaging modalities. Hence, a stable coronary lesion may determine an impairment to coronary reserve, and, in the presence of other coexisting conditions, both the direct and indirect impact of catecholamines on cardiac cells and coronary vessels may cause a reduction in cardiac output that accompanies transient heart dysfunction, which might jeopardize perfusion of the coronary system and ultimately result in more pronounced ischemia and myocardial stunning.

On the other hand, the second possible scenario is that TTS develops during the occurrence of ACS. The underlying processes involved in ACS, including coronary artery spasm, elevated heart oxygen consumption, intensified platelet response, and even plaque rupture, may favor the development of TTS. Moreover, severe pain, such as that seen in ACS, is a recognized TTS trigger [77].

The third and rarest condition that may occur is acute coronary syndrome as a consequence of TTS [78]. As discussed later, this reciprocal cause-and-effect relationship between CAD and TTS is still under discussion.

However, discovering the underlying mechanisms of the eventual CAD–TTS association on an individual basis can be challenging and difficult, especially in the acute setting.

## 5. Takotsubo Triggered by Ischemia—The Chicken-Egg Paradox

An intriguing condition is TTS caused by ACS, identified when new myocardial wall abnormalities are present and not correlated with the obstructive coronary vessel. TTS caused by ACS has been reported in a few published cases [79]. It has been proposed that patients with TTS may experience myocardial stunning because of transitory ischemia brought on by plaque rupture followed by fast lysis [1]. The rupture, fissuring, or erosion of an atheromatous plaque and subsequent thrombosis is a recognized trigger mechanism for intracoronary thrombosis. During the early stages of ACS, spontaneous intermittent coronary recanalization and reocclusion are common, and this repetitive variable combination of both phenomena may provoke myocardial stunning and akinesia of the myocardium, particularly in coronary arteries supplying a large LV territory [80]. In fact, coronary angiography results constituted the primary basis for previous criteria that suggested the absence of obstructive CAD for the diagnosis of TTS. However, intravascular imaging may be a more useful tool in characterizing patients at risk and explaining the association between CAD and TTS, given the low diagnostic performance of coronary angiography in identifying the aforementioned conditions [37].

Clinical manifestations, evolving ECG abnormalities, and echocardiographic findings of post-ischemic myocardial stunning and TTS are nearly identical due to microvascular dysfunction, multiple-vessel coronary spasm, and an abortive myocardial infarction in a long wrap-around left anterior descending artery [77]. Moreover, also in the setting of acute myocardial ischemia, as observed in TTS, there is a significant neuro-hormonal activation, which includes norepinephrine spillover, severe sympathetic nervous system activation with local cardiac sympathetic disturbance, and catecholamine cardiac toxicity [77,79]. This last phenomenon is elevated explicitly in peri-infarct zones, becoming hyperkinetic and hyper-contractile, possibly as a result of elevated catecholamine levels [81]. All these findings explain why acute myocardial infarction correlates with TTS more often than other somatically stressed disorders [82].

A considerable percentage of individuals with STEMI and culprit lesion in the LAD artery can exhibit a TTS alteration pattern [79]. Indeed, TTS caused by ACS has been shown to involve primarily the LAD artery. However, there are cases where flow-limiting lesions are present in other vessels, such as in the obtuse marginal branch or right coronary artery, that may also trigger TTS [83,84]. Moreover, while TTS patients with LAD stenosis generally present a classical apical ballooning pattern, alternative localization of the coronary lesion, such as in the obtuse marginal or diagonal branch, was seen to play a role in the development of the mid-ventricular type of TTS [79]. This suggests that the specific localization of the CAD may correlate with and contribute to developing different TTS variants. However, the absence of significant obstruction during angiography does not exclude TTS driven by ischemia in certain situations. Favorable remodeling of lipid-rich plaques that may be associated with temporary clot formation and spasm may go undetected by coronary angiography [84]. Thus, one should regard a potential LV dysfunction and wall motion abnormalities that resemble TTS to LV stunning caused by temporary occlusion of the LAD as components of the normal atherosclerotic pathway [85]. In this setting, intravascular imaging may play a significant role during coronary angiography. Indeed, OCT demonstrated a significant prevalence of extremely vulnerable thin cap fibroatheromas prone to rupture and thrombosis in TTS patients [38]. Moreover, in a series by Ibanez et al., including patients with TTS, the authors found a single, ruptured atherosclerotic plaque in the middle portion of the LAD artery using IVUS, suggesting that the underlying cause of this pathological condition might be an ACS with early reperfusion, less enzymatic release, and LV stunning rather than necrosis [37]. Notably, all described cases had a well-developed LAD, with a long course around the left ventricle apex, supplying a large myocardial zone compatible with the typical akinetic abnormalities of TTS, also suggesting an anatomical predisposition for this syndrome [37].

Furthermore, acute ischemia from sudden coronary artery dissection (SCAD) can also cause an ischemic process, potentially resulting in acute post-ischemic myocardial stunning (PIMS) [86]. Therefore, TTS has also been observed to be driven by myocardial infarction following SCAD, especially in the LAD artery or diagonal branch [79]. According to this, TTS was proposed to be a second name for PIMS. In fact, like any additional physical stressor, the ischemia insult brought on by SCAD may contribute to the development of myocardial stunning by causing local heart sympathetic disruption, sympathetic hyperactivation, norepinephrine seethe, and spillover [87]. However, neurohormonal, genetic, anatomical, and other variables may interact in a complicated way to contribute to the pathophysiology of TTS in SCAD [87].

**Figure 2:**
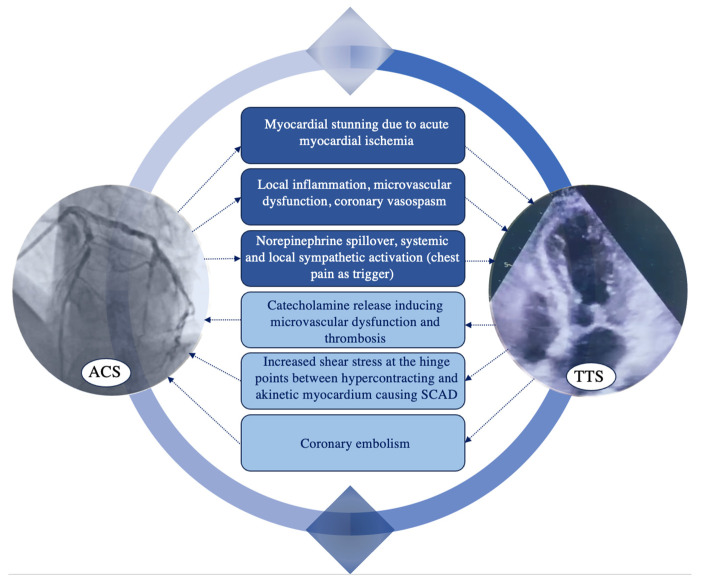
Bidirectional cause–effect relationship and underlying mechanisms between coronary artery disease and Takotsubo syndrome. ACS = acute coronary syndrome; SCAD = spontaneous coronary artery dissection; TTS = Takotsubo syndrome.

Even though there are more data about the ischemia-driven TTS, primarily secondary to ACS, there is some evidence of ACS secondary to TTS [88]. The bidirectional cause–effect relationship between CAD and TTS is illustrated in Figure 2. 

ACS can happen even in the initial hours following TTS, particularly in cases showing a post-procedural low TIMI flow grade due to TTS-related microvascular dysfunction [88]. Moreover, TTS could also cause SCAD as a result of increased mechanical stress at the hinge point between hyper-contracting segments and the TTS-induced akinetic stunned myocardium [89]. However, the most plausible explanation of ACS induced by TTS is based on the high catecholamine levels in this setting that have been demonstrated to have prothrombotic properties in coronary arteries. Within 1–2 days following the onset of symptoms, the levels of catecholamines in TTS patients are nearly three times greater than those in myocardial infarction patients and 20 times higher than those in normal individuals [90]. Lin and Young showed that the occurrence of cyclic blood flow decreases in the canine coronary arteries grew by 60% when the plasma epinephrine concentration was raised to about 27 nmol/L [91]. Given that catecholamines are known to have prothrombotic effects and that TTS is linked to an increase in catecholamines, it is plausible that TTS preceded ACS in certain case reports that have been documented in the literature [78]. Despite coronary artery thrombosis, SCAD, or LV systolic dysfunction as potential pathological mechanisms, ACS in this setting might sometimes be secondary to embolic events from a LV thrombus developed in the akinetic LV apex. 

No matter the still ongoing debate about the chicken–egg paradox underlying the relationship between CAD and TTS, it is important to consider both conditions in order to choose the right diagnostic and therapeutic strategies.

## 6. Takotsubo and Coronary Syndromes—To Differentiate or Co-Treat

### 6.1. Diagnostic Approach for Differentiation of CAD and TTS

Even while the majority of clinicians nowadays are aware that patients with TTS may also have concurrent CAD, it can be challenging to distinguish between these two conditions in a given patient. The recent advances in intra-coronary and cardiac imaging techniques have significantly facilitated the distinction between TTS and a recent ACS since OCT-based intracoronary imaging could easily verify the existence or absence of a ruptured plaque. In contrast, cardiac magnetic resonance (CMR) can definitively rule out the presence of late gadolinium enhancement (LGE), that could indicate myocarditis or myocardial infarction [92]. However, these techniques may be inappropriately time-consuming, especially in the acute setting, and require economic resources that are not available everywhere. On the other hand, a multimodality imaging strategy involving echocardiography, CMR, single photon emission computed tomography (SPECT) or PET might sometimes be necessary for the diagnosis of TTS, as coronary angiography and left ventriculography alone are not sufficient for this purpose. Main electrocardiographic (ECG), laboratory, and imaging parameters used in the differential diagnosis between TTS and ACS are summarized in Table 1. However, despite recent advancements in imaging modalities, distinguishing between TTS and a true aborted MI without lasting cardiac damage remains difficult [93].

Moreover, several clinical and ECG parameters may be considered to guide the differential diagnosis between TTS and CAD. Sometimes, a combination of a few of them can be useful. The International Takotsubo Registry reasoned that the combination of seven factors (female sex, emotional or physical stress, ST depression, psychiatric or neurological disorder, as well as QTc prolongation) would produce a potent predictive score for the diagnosis of TTS [94]. Using this tool, TTS diagnosis is supported by a score higher than 70 points. On the other hand, in order to rule out acute plaque rupture, a score of less than 70 would require an angiographic evaluation [95].

### 6.2. Biomarkers

Patients with TTS often have less tissue necrosis or none, which results in lower levels of cardiac troponin peak and creatine kinase–MB (CK–MB) compared to patients with STEMI. However, upon admission, troponin values in TTS usually do not differ from those in AMI [96]. The troponin/CK–MB ratio has been found to be a suitable parameter that can discriminate TTS from AMI [97]. Moreover, TTS is generally associated with elevated levels of neuropeptide-Y, serotonin, and plasma catecholamines (epinephrine, norepinephrine, and dopamine), as demonstrated in several studies [90,96]. Apart from the recognized indicators, several innovative markers have been suggested to differentiate TTS from AMI. In this regard, soluble suppression of tumorigenicity 2 (sST2) and soluble thrombomodulin (sTM) plasma concentrations could differentiate between patients with acute anterior STEMI and those with TTS [98]. In addition, circulating microRNAs such as miR-26a, miR-1, miR-133a, and miR-16, along with growth differentiation factor-15 (GDF-15), could distinguish between ACS and TTS [99,100]. However, physicians should not forget that serum levels of N-terminal pro–b-type natriuretic peptide (NT-proBNP) are also increased in TTS and their measuring can be useful for monitoring cardiac damage and recovery [101]. Some evidence propose that a distinct cardiac biomarker profile, which is marked by a sharp rise in NT-proBNP during the first few days in the presence of only slightly elevated indicators of myocardial necrosis (NT-pro BNP/troponin ratio), may be used to distinguish TTS from ACS [101]. 

### 6.3. Electrocardiographic Changes

ST-segment elevation and T inversion, present in nearly 40% of the patients and primarily in the precordial leads, are common ECG abnormalities in TTS patients [102]. Therefore, making a differential diagnosis in the acute setting can be very challenging. Kosuge et al. compared the ECG findings of patients with TTS or STEMI admitted within 6 h of the beginning of symptoms; they found that TTS could be diagnosed with 91% sensitivity and 96% specificity when lead aVR showed ST-segment depression and lead V1 showed no ST-segment elevation [103]. Additionally, the lack of reciprocal alterations in inferior leads, the absence of abnormal Q waves, and the decreased occurrence of ST-segment elevation of ≥1 mm in lead V1 support TTS [104,105]. On the other hand, transient lengthening of QTc (often greater than 500 msec) is a common ECG occurrence in TTS and considerably less common in AMI, which recovers to normal during the recovery phase [106]. Given that 60% of TTS patients experience QT interval prolongation after 72 h, dynamic alterations in the patients’ ECGs are common. Serial ECGs must, therefore, be performed from the time of admission until 72 h later [107]. Furthermore, amplitude attenuation of the QRS complexes in serial ECGs and low QRS voltage in the admission ECG are very common manifestations in TTS patients. Indeed, in the absence of pericardial effusion, it has been suggested that myocardial edema may be the source of these ECG abnormalities, which could assist TTS diagnosis and help distinction from ACS [108].

### 6.4. Echocardiography

The primary imaging technique for evaluating changes in ventricular function, including pericardial effusion, presence of LV thrombi, hemodynamic state, right ventricle (RV) involvement, regional wall motion abnormalities (RWMA), and systolic pulmonary artery pressure evaluation, is echocardiography [109]. However, differences between ACS and TTS can be difficult to explore using echocardiography. Firstly, echocardiography can be used to identify several variants of the classical apical ballooning syndrome, such as midventricular TTS, characterized by hypo-, a-, or dyskinesia of the midventricular segments, basal forms, and focal TTS primarily involving the anterolateral region [95]. Moreover, right ventricular dilatation with hypo- or akinesia of the free wall and apex in its solitary form might occasionally be used to diagnose RV engagement [110]. Interestingly, wall motion anomalies are not limited to a single coronary artery vessel in any TTS type except for the focal variant [60].

Up to 12.5–25% of cases with TTS may have left ventricular outflow tract (LVOT) obstruction, which is identified by echocardiogram (significant if peak instantaneous LVOT gradient ≥ 30 mmHg). It may be caused by basal hypercontractility with or without systolic anterior motion (SAM) of the anterior mitral leaflet, resulting in dynamic obstruction of the LVOT [111]. SAM motion can cause leaflet malcoaptation, which can lead to secondary mitral regurgitation (MR) in addition to the dynamic intraventricular obstruction. The timely identification or exclusion of LVOT obstruction is essential for regulating patient management and preventing the administration of unsuitable treatments that may exacerbate a hemodynamic state, as described later [109]. 

Additionally, 2–8% of TTC patients have thrombus in the akinetic ventricular apex, which can occasionally result in an arterial embolism or stroke [112,113]. It is important to identify LV thrombus by echocardiogram before the clinical and therapeutic planning. Indeed, in case of LV assist device implantation necessity, LV thrombus is a relative contraindication [114]. Moreover, it necessitates anticoagulation therapy that may influence the treatment decision about eventual coexisting CAD. Therefore, echocardiography constitutes the first-line imaging modality, that should possibly be performed before coronary angiography, in order to assess alterations in left and right ventricular function and motion, hemodynamic status, mechanical complications such as LVOT obstruction, presence of LV thrombi, and pericardial effusion [109].

However, compared to patients with ACS, those with TTS have substantially reduced systolic left ventricular function [51]. Moreover, in a recent study comparing TTS patients to STEMI patients, the TTS group showed a lower LVEF and a higher wall motion score index (WMSI; 1.98 + 0.2 vs. 1.51 + 0.14; *p*, 0.001) [115]. In addition, the number of areas with RWMA ≥ 4 and a WMSI value ≥ 1.75 predicted TTS with a sensitivity of 83% and 84% and a specificity of 100% and 97%, respectively [115]. Nevertheless, in some cases, advanced echocardiographic parameters may be useful. The impairment of global longitudinal strain, untwist rate, and time to peak untwisting have been found to continue for weeks to months following acute TTS and can be additional supporting metrics [116]. Nevertheless, it was well recognized that a single coronary event is a highly implausible explanation for the observed ventricular dysfunction when RWMAs extend into additional coronary artery distributions, such as in the setting of TTS [59]. Even so, some authors have contested this strategy, claiming that more research needs to be done on the LAD distribution to support the idea that RWMAs found in TTS extend beyond the LAD’s limits [85]. Tomographic imaging modalities, such as nuclear imaging or CMR, might be better in this sense. 

### 6.5. Cardiac Magnetic Resonance

CMR is the gold standard for accurately quantifying the volumes and functions of the LV and RV and for qualitatively assessing RWMA. It enables a thorough assessment of structural and functional anomalies in TTS patients [117]. When employing T2 weighted imaging to detect edema in areas with aberrant systolic function, it is possible to evaluate the acuteness, severity, and magnitude of myocardial stunning among individuals with TTS. This is especially useful during the acute phase, as the edema goes away in conjunction with the LV function recovery in a few weeks [117]. Moreover, cardiac tissue characterization is a significant benefit of the CMR due to its capacity to distinguish between reversible and irreversible myocardial damage. The lack of LGE in dysfunctional left ventricular segments of patients with TTS makes it possible to distinguish between TTS and AMI that shows transmural or subendocardial LGE associated with a specific coronary distribution region [117,118]. Moreover, it can help differentiate TTS from the majority of cases of acute myocarditis that typically show epicardial or patchy LGE [118]. According to these findings, lately, distinct CMR criteria were developed for diagnosing TTS during the acute phase including the presence of edema, typical RWMA, and the lack of irreversible tissue destruction seen by LGE [95]. However, these criteria cannot be applied universally to all patients, for several reasons. Firstly, while large regions of LGE unmistakably favor the diagnosis of AMI, the absence of LGE, which can also be missing in a small percentage of patients with ACS, does not enable diagnosing TTS in all individuals [119]. In fact, nearly 40% of patients with CAD and concomitant TTS can show the presence of LGE [120]. In addition, patients with TTS have shown modest focal or patchy LGE when low LGE signal intensity thresholds of three standard deviations (SD) are used. In contrast, no areas of LGE were observed in TTS patients when signal intensity thresholds of five SDs above the mean were considered [117]. Lastly, an additional parameter can be included, according to a recent study. Compared to anterior STEMI, CMR imaging revealed a markedly reduced left atrial function during the acute/subacute phase of TTS [121]. 

Notably, using CMR in patients with CAD and TTS can be difficult in the acute setting. Therefore, in clinical practice, most patients instead undergo coronary angiography early after admission into the emergency department. 

### 6.6. Coronary Angiography and Coronary Physiology Assessment

TTS and CAD commonly show an identical clinical presentation, and in the case of a patient with suspected CAD, a coronary angiography should be performed according to guidelines [122]. However, the degree and pattern of acute LV dysfunction cannot be well explained by stenoses alone, which may or may not be hemodynamically significant in many patients with bystander coronary disease [123]. Therefore, a comprehensive analysis of left ventriculography and angiography is necessary to evaluate the possibility of a perfusion–contraction mismatch [53].

Desmet et al. showed that approximately one-third of patients with characteristic apical ballooning exhibit a tiny region in the most apical portion of the LV with preserved contractility. This sign, known as the “apical nipple sign,” is useful in differentiating between anterior STEMI, which lacks this sign, and TTS [124,125]. Additionally, in individuals suspected of having TTS, SCAD should be considered as a differential diagnosis. Coronary angiograms should be performed for SCAD, as TTS and SCAD may coexist [126]. In situations like the present one, intravascular imaging techniques such as IVUS or OCT can be helpful since they can identify additional forms of myocardial infarction and no obstructive coronary artery disease (MINOCA) [38]. 

The pathophysiology of TTS is significantly influenced by CMD, and coronary physiologic indicators are thought to be the fundamental tools for comprehending the physiological evaluation of coronary blood flow in TTS.

The coronary flow and coronary microcirculation measures that are typically examined are the index of microcirculatory resistance (IMR; abnormal if >25), coronary flow reserve (CFR; abnormal if <2), and coronary fractional flow reserve (FFR; substantial coronary lesion if FFR < 0.8). While IMR particularly examines the microcirculation’s status and is not dependent on the presence of epicardial stenosis, CFR is a valuable indicator of the microcirculation’s state and is impacted by epicardial artery lesions [127]. Moreover, a few studies, mostly case reports, have demonstrated that TTS patients have reduced CMR and increased IMR values, which support microvascular dysfunction, in the presence of normal FFR values, which rule out occult epicardial coronary lesions [128,129]. In a recent study, Solberg et al. reported that IMR values above 25, indicating microvascular dysfunction, were found in nearly a third of TTS patients in the acute phase [130]. Similarly, Castaldi et al. demonstrated that CMD, as measured by angiography-derived IMR, was inversely linked with LV function and was a common finding in TTS [131]. Interestingly, there is a correlation between an increase in LV function and a temporal improvement in microvascular resistance [129]. CMD is more common in the acute period of TTS patients, even when compared to age- and gender-matched patients with ischemia and no obstructive coronary artery disease (INOCA) [132]. Moreover, the TTS’s apical phenotype exhibits a greater degree of CMD than its midventricular phenotype [132]. However, using coronary physiology for differential diagnosis is challenging, as both coronary syndromes and TTS present microvascular dysfunction. Indeed, a recent study demonstrated that microcirculatory function is compromised in TTS patients during the acute phase, and patients with TTS have microvascular dysfunction as high as those with STEMI, as determined by IMR [133]. These findings suggest that, despite their distinct settings, both groups may experience similar microvascular damage. However, it is important to underline that according to the most recent European guidelines for management of acute coronary syndrome, invasive epicardial functional assessment, even of non-culprit segments of the infarction-related artery, is not recommended during the acute phase of the ACS [122]. On the other hand, CMD is known to mitigate pharmacologically produced myocardial hyperemia in the acute phase of TTS, which may confuse the FFR-based functional assessment of coronary stenosis [134]. Therefore, early FFR measurement of coronary stenosis is not an accurate procedure when concomitant CAD and TTS are present.

Alternative parameters, such as hyperemic stenosis resistance (HSR) and hyperemic microvascular resistance (HMR) are less widely used in clinical practice. Only one case report described normal HSR after adenosine infusion and high HMR (>2), especially in the LAD coronary artery with a greater proportional response to adenosine in a patient with TTS [135]. 

Moreover, thrombolysis in myocardial infarction (TIMI) corrected frame count (cTFC) is a measure for coronary flow evaluation with a possibility to detect an increase in microvascular resistance when a coronary slow flow pattern is present [136]. According to a recent study that used this approach, TTS had a much higher cTFC in the LAD artery than did controls, and this finding may help to explain the severe apical involvement [137]. However, the implementation of this method in the TTS assessment is difficult, as it is influenced by clinical, technical and physiological factors [138]. To clarify the function of coronary physiology assessment in TTS and outline its use in clinical settings more research is needed.

### 6.7. Ventriculography and Hemodynamics

Patients with TTS first show anomalies in their heart hemodynamics, but little is known about the ventricular dysfunctions that accompany this condition. The assessment of LV end diastolic pressure (LVEDP) and the presence of a pressure gradient in the outflow tract can be done through invasive hemodynamic evaluation [139]. It is imperative to do cardiac catheterization, which involves inserting a fluid-filled catheter into the left ventricle to record LV pressures, and left ventriculography, which involves using a pig tail catheter, in all patients with TTS, unless there is a contraindication. Indeed, if left ventriculography is not contraindicated, it should be a part of the invasive evaluation of TTS patients, not only to establish the wall motion alteration profile, but to quickly exclude a concomitant mitral regurgitation, which carries valuable prognostic information [124]. In addition, a careful investigation of the presence of LVOT obstruction, which may affect 20% of TTS patients, should be conducted during left catheterization when the pigtail catheter is retracted from the LV apex beyond the aortic valve [95]. LVOT obstruction in TTS has important prognostic implications, as it may lead to hemodynamic instability and cardiogenic shock and carries implications for treatment, as described later [140]. Thus, one of the most challenging consequences that can arise during the acute phase of TTS is LVOT obstruction, whose severity varies based on hemodynamic condition. Assessing LVEDP has in fact been demonstrated to be a valid predictor of in-hospital complications for TTS patients [141]. Utilizing a conductance catheter inserted into the LV of TTS patients, a very recent study known as the OCTOPUS (Optimized Characterization of Takotsubo Syndrome by Obtaining Pressure Volume Loops) trial analyzed pressure–volume loops for assessment of systolic and diastolic load-independent function. This study provided detailed information regarding cardiac energetics and efficiency as well as ventricular–arterial coupling. The trial results indicate that TTS is linked to prolonged active myocardial relaxation, poor myocardial energy usage, a shortened systolic duration, and significantly reduced cardiac contractility, while diastolic passive stiffness is unaffected. In order to maintain the stroke volume, the heart responds by expanding the LV end diastolic volume. These results could point to reduced myofilament protein phosphorylation as a possible treatment target for TTS [142]. However, differential diagnosis may be challenging as TTS and ACS both present severe diastolic dysfunction, high diastolic pressures, and equally reduced systolic function [143].

### 6.8. Other Imaging Modalities

Several other imaging modalities such as SPECT and PET might be helpful in the differential diagnosis between TTS and ACS, but they are not frequently used in clinical practice. Normal or mild reduction of perfusion in dysfunctional segments using myocardial perfusion scintigraphy and reduced metabolic activity in the impaired regions during SPECT with 123I-b-methyl-iodophenyl pentadecanoic acid and PET using 18F-flourodeoxyglucose (FDG) are further support for TTS diagnosis [95,123]. SPECT imaging of the cardiac absorption of 123I-metaiodobenzylguanidine (123I-MIBG) reveals the sympathetic innervation of the heart. In dysfunctional segments, 123I-MIBG is decreased for months while perfusion is nearly normal, which is compatible with a local disruption of sympathetic neural activity. In the subacute setting, combining SPECT perfusion imaging with 123I-MIBG SPECT allows one to rule out ACS, which is characterized by decreased innervation and perfusion [144].

## 7. CAD and Takotsubo Synergism—Possibility of Cotreatment

Even though differential diagnosis can be important to stabilize the priority of the treatment, it is also essential to keep in mind that CAD and TTS can frequently coexist, necessitating a different point of view in the therapeutic strategy. In contrast to AMI, which requires immediate restoration of the blood flow through revascularization, TTS is treated mainly by removing the component generating physical or psychological stress and avoiding potential life-threatening conditions (such as arrhythmias or CS) [95]. Following the acute phase of the TTS, patients can experience significant rates of mortality (5.6% of deaths per patient-year) and complications (1.7% of strokes or transient ischemic attacks per patient-year). Therefore, it is particularly relevant to determine if medical intervention affects the course of TTS following its initial phase [51]. 

Even though there is an urgent demand to treat individuals with chest discomfort exhibiting ST-segment elevation, TTS must be distinguished from AMI, if not concomitantly, as soon as possible, in order for the patient to receive the proper treatment. According to the Chest Pain Center model, the intense preoperative discussion surrounding percutaneous coronary intervention (PCI) and the urgent operation process for AMI can worsen a patient’s psychological state, increasing the release of catecholamines which ultimately aggravates the progression of TTS [21]. On the other hand, ACS, if not promptly recognized and misdiagnosed as TTS, can be fatal. Therefore, the prehospital emergency physician must differentiate between AMI and TTS. 

However, CAD and TTS can frequently coexist, as discussed above, and both conditions should be treated simultaneously, choosing the most appropriate strategies for each patient, and trying to avoid using specific therapies that may worsen one of these two conditions. In TTS patients, concomitant CAD is often “neglected” and undertreated. As previously mentioned, even in the case of a non-obstructive CAD and TTS, physicians should always investigate the presence of a SCAD [87]. Therefore, intravascular imaging should be used in these settings. On the other hand, it can be life-threatening to fail to recognize TTS in an ACS patient since recurrent TTS has been found to show clinically differently and with varying patterns of LV involvement [145]. Treatment with watchful waiting is advised for stable TTS patients with SCAD who have a patent coronary artery. In contrast, PCI should be considered in unstable patients with cardiogenic shock and flow-limiting coronary lesions. However, it should be kept in mind that cardiogenic shock may be caused by the related TTS, particularly in cases with LVOT obstruction, and not by the SCAD [89]. Moreover, TTS may exacerbate AMI and occasionally lead these individuals into CS. Hence, creating techniques for reversing TTS could aid patients who arrive with CS driven by AMI [82].

Supportive therapies, first with diuretics and vasodilators, are the basis of management during the acute phase of TTS, which is still empirical [95]. Angiotensin-converting enzyme inhibitors or angiotensin-receptor blockers were shown to be associated with better survival in TTS patients. In contrast, beta blockers reduce the risk of arrhythmias and may prevent TTS recurrences [51,146]. Both classes of drugs are also indicated in the medical long-term treatment of ACS patients [122]. Moreover, if concomitant coronary atherosclerosis is present, aspirin and lipid-lowering therapies are appropriate for both conditions [95]. Furthermore, ACS is more frequently associated with hypertension that requires treatment with nitrates [147]. On the other hand, when TTS is present, administration of nitroglycerin in the presence of LVOT obstruction has been found to worsen the pressure gradient and, therefore, should be avoided [95]. Moreover, using catecholaminergic drugs may be helpful in patients whose ACS is exacerbated by CS [148]. However, as catecholamine inotropic drugs have been linked to a 20% increased risk of death, it is best to avoid them when there is concurrent TTS [51]. In this setting, levosimendan, a calcium sensitizer, has been proposed as a safe and efficient substitute for catecholamine drugs in complicated TTS [149]. 

Mechanical circulatory support should be taken into consideration for patients who are refractory to cardiogenic shock [150]. Regarding TTS-related CS, the use of Impella and venoarterial extracorporeal membrane oxygenation (ECMO) as mechanical circulatory support might be considered [151]. Similarly, patients with CAD and CS, as well as those undergoing complex PCI who have significant LV dysfunction undergo Impella device positioning more frequently nowadays [114]. However, prospective studies are necessary to evaluate the efficacy and safety of different devices and to find the best time to start mechanical circulatory support in this special population of TTS and CAD patients. 

**Figure 3:**
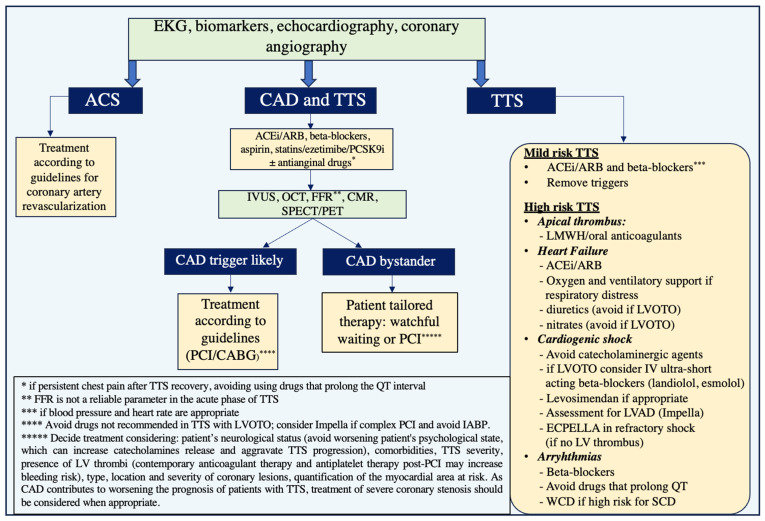
Proposed algorithm for choosing the most appropriate diagnostic methods and therapeutic strategy in patients with Takotsubo and coronary artery disease. ACEi = Angiotensin-converting enzyme inhibitors; ACS = acute coronary syndrome; ARB = Angiotensin II receptor blockers; CABG = coronary artery bypass graft; CAD = coronary artery disease; CMR = cardiac magnetic resonance; ECPELLA = Extracorporeal Membrane Oxygenation–Impella; FFR = Fractional flow reserve; IV = intravenous; IABP = Intra-aortic balloon pump; IVUS = Intravascular ultrasound; LMWH = Low molecular weight heparin; LV = left ventricle; LVAD = Left ventricular assist device; LVOTO = Left ventricular outflow tract obstruction; OCT = Optical coherence tomography; PCSK9i = proprotein convertase subtilisin/kexin type nine inhibitor; PCI = percutaneous coronary intervention; PET = Positron Emission Tomography; SCD = sudden cardiac death; SPECT = Single Photon Emission Computed Tomography; TTS = Takotsubo syndrome; WCD = wearable cardioverter-defibrillator.

Furthermore, treatment of concomitant coronary lesions remains debatable. Deciding whether CAD is a trigger of the TTS, or a simple bystander, may influence the treatment plan decision, as illustrated in Figure 3. 

Indeed, the coexistence of CAD and TTS makes treatment planning challenging. In this setting, intravascular imaging using IVUS or OCT, FFR measurement, as well as CMR or SPECT/PET may be useful in the characterization of coronary lesions and assessment of their direct or indirect connection with the coexisting TTS. It is crucial to emphasize that microvascular dysfunction might counteract the effects of pharmacologically produced myocardial hyperemia, which can confound the functional evaluation of coronary stenosis. Thus, FFR is not a trustworthy metric in the acute context of TTS, and it may be necessary to repeat catheterization with functional evaluation at least one month following the acute phase [134].

As previously discussed, catecholamines may have prothrombotic effect, and in patients with TTS the higher levels can induce stent thrombosis [152]. Therefore, it is unknown whether stenting of bystanders but significant coronary artery stenosis in the acute setting of TTS can worsen the risk of stent failure. Some authors suggest total revascularization in cases of severe CAD with critical stenoses, putting a significant myocardial area at risk (such as critical left main lesions and/or severe multivessel CAD) when TTS is linked to life-threatening hemodynamic instability [153]. However, in the lack of studies, patients with concomitant coronary artery disease should be treated according to the current guidelines [122]. Surely, choosing the best course of action and preventing mistreatment requires an accurate detection of the coexistence of the two scenarios and evaluation of potential complications [89].

## 8. Conclusions

The paradigm change has prompted additional research into the possible connection between TTS and CAD, while increasing clinical and research experience have gradually challenged the notion that these two disease states are mutually exclusive. The exact underlying pathophysiology of TTS is still unknown, even though novel processes such as inflammation, metabolic alteration, and microvascular dysfunction have been suggested to regulate the disease’s development and its association with CAD. According to recent studies, ACS can trigger TTS, and TTS may occasionally result in secondary ACS. Although the exact nature of the association between CAD and TTS is yet unknown, misdiagnosis of the two conditions may result in patients receiving suboptimal therapy that sometimes may carry detrimental consequences. Therefore, making a differential diagnosis or confirming their coexistence using laboratory assays, imaging, and interventional techniques is challenging. However, choosing the most appropriate medical or percutaneous treatment on an individual basis remains crucial. Thus, it is necessary to conduct additional research to determine if patients with TTS should receive staged or ad hoc PCI. Although other imaging techniques, such as CMR, may not precisely illustrate the relationship between the two coexisting conditions, greater application of intracoronary imaging might seem appropriate given their common coexistence and the intricate connection between CAD and TTS.

## 9. Future Directions

TTS is a challenging acute cardiac disorder that resembles AMI and is correlated with a variety of pathophysiological mechanisms that are not mutually exclusive. Since both conditions may occur simultaneously, diagnosis might be more difficult, particularly in emergency situations. Thus, further research into straightforward, sensitive, and specific testing methods would considerably streamline clinical care pathways and reduce the risk of misdiagnosis. Within the spectrum of stress cardiomyopathy, TTS with CAD could fit into a unique category with a unique diagnostic and treatment plan to adhere to. Furthermore, the relationship between CAD and TTS and their potential bilateral causal effect may be explained by the development of innovative intravascular ultrasound and molecular techniques, as well as parameters for an invasive functional assessment of coronary microcirculation. This might establish a basis for upcoming systemic and focused therapy randomized trials. Similarly, new approaches such as anti-inflammatory, coronary microvascular dysfunction, and sodium-glucose transporter-2 inhibitor therapy seem promising and might eventually be implemented in the management of TTS with CAD. To clarify the pathophysiological process and the most appropriate treatment for patients with TTS and CAD, more randomized trials are necessary.

## Figures and Tables

**Table 1:** Electrocardiographic, laboratory, and imaging parameters used in the differential diagnosis between Takotsubo syndrome and Acute coronary syndrome.

	Takotsubo Syndrome	Acute Coronary Syndrome
***Laboratory***	High NT-proBNP levels Moderate cTnI elevation high NT-proBNP/cTnI ratio microRNAs/sST2, STM	cTnI above 99° percentile High CK-MB levels Low NT-proBNP/cTnI ratio
***ECG***	ST depression in aVR ST elevation and T wave inversion in precordial leads with no reciprocal alterations No ST elevation in V1 lead Dynamic QTc prolongation	Regional ST-segment and reciprocal alterations T wave inversion Arryhthmias
***Echocardiography***	Transient and greater LV dysfunction Apical ballooning/ midventricular, basal or focal variants Motion wall anomalies not limited to a single coronary vessel LVOT obstruction WMSI ≥ 1.75 Involved RV wall motion anomalies	Normal or persistently (but less impaired than TTS) LV dyfunction Usually limited regional wall motion alterations
***Cardiac magnetic*** ***resonance***	Elevated T1 and T2 values Generally, absence of LGE (or modest focal or patchy LGE) Typical patterns of wall motion alterations Reduced left atrial functioning	Transmural or subendocardial LGE Regional wall motion impairment
***Coronary angiography/ventriculography***	Normal, non-obstructive or obstructive coronary artery disease Apical nipple sign Perfusion-contraction mismatch	Obstructive coronary artery disease
***Hemodynamics***	LVOT obstruction diastolic dysfunction, severe systolic dysfunction High LVEDP	Diastolic dysfunction Moderate/severe systolic dysfunction
***Other imaging techniques***	SPECT/PET and SPECT with MIBG: normal or mild reduction of perfusion, long-term decreased innervation in dysfunctional segments	SPECT/PET: significant reduction of perfusion

Abbreviations: CK–MB = creatine kinase–myocardial band; LGE = Late gadoliniu Enhancement; LV = left ventricle; LVEDP = left ventricular end diastolic pressure; LVOT = Left Ventricular Outflow Tract; MIBG = metaiodobenzylguanidine; NT-proBNP = N-terminal pro–B-type natriuretic peptide; cTnI = cardiac troponin I; PET = Positron Emission Tomography; RNAs = ribonucleic acids; RV = right ventricle; sST2 = soluble suppression of tumorigenicity; STM = soluble thrombomodulin; SPECT = Single Photon Emission Computed Tomography; TTS = Takotsubo syndrome; WMSI = wall motion score index.

## Data Availability

Not applicable.
